# The 4 Ss Framework for Designing Age-Friendly Communities of the Future: Focus Group Study

**DOI:** 10.2196/90923

**Published:** 2026-09-25

**Authors:** Inga Hunter, Caroline Lockhart

**Affiliations:** 1 School of Management and Marketing, Massey Business School, Massey University, Business Studies Complex, 3rd floor, Tennent Drive, Palmerston North, Manawatu-Wanganui, 4442, New Zealand, 64 6 356 9099 ext 84913

**Keywords:** older adults, digital literacy, digital health, social connection, social isolation, well-being, trust, technology adoption, rural health services, health equity

## Abstract

**Background:**

Digital technology is increasingly being used to deliver interventions and initiatives to support the well-being of older adults. However, few studies have conducted needs assessments to identify the future well-being service requirements of an older adult population and their preferred modes of delivery, whether via digital technology or, in- person, or a combination of both (ie, a hybrid model).

**Objective:**

This study aims to investigate the requirements of a rural region in New Zealand to inform planning to meet the future well-being needs of its older adult population over the next 30 years.

**Methods:**

In total, 2 focus group discussions and 13 interviews were held with participants using a combination of phone and video calls. A total of 31 adults aged 61 years or older participated. The participants were asked how they saw the future well-being needs of the older adult population evolving, the role of digital technology and/or in-person interactions in delivering well-being services, and perceived barriers to, and enablers of, digital technology for providing services. Focus group and interview transcripts were thematically analyzed.

**Results:**

A total of 4 key well-being themes were identified across both focus group discussions and interviews with participants: —“services,” “support,” “spaces,” and “social connection,” —with an overarching finding that future age-friendly planning must prioritize social and community well-being outcomes, ensuring that investments in services, digital inclusion, and public spaces strengthen older adults’ ability to connect with others and participate fully in community life.

**Conclusions:**

Digital technology is developing at a rapid pace, and as a result, we need to consider how to plan for the transition and bridge the gap identified between the current use of digital technology and its potential future use if technology is to support the older adults of the future. The findings indicate that older adults prefer to engage in- person, while trust is a barrier to digital technology use for some participants. The future offers many opportunities to support the well-being of older adults and communities through the application of the proposed 4 Ss Framework.

## Introduction

### Background

The World Health Organization (WHO) frames health as a dynamic resource that enables people to meet their needs and aspirations [1]. This broad understanding recognizes that health and well-being are shaped by the wider environment in which people live, and many determinants of health exist outside of the traditional scope of the health sector [2]. The WHO applied these determinants to the health and well-being of older adults to promote the concept of “active aging” through its Active Aging Framework [3]. The Healthy Ageing Strategy in New Zealand [4] considers that older people should live well and age well in “age-friendly” communities, thus linking active aging with Age-Friendly Cities and Communities (AFCC).

Van Hoof et al [5] developed their model of the age-friendly city with 8 domains that align with the WHO dimensions of active aging: outdoor spaces and buildings;, transportation;, housing;, civic participation and employment;, respect and social inclusion;, social participation;, communication and information;, and community support and health services. This is very similar to the 5 key areas identified by the Office of the Seniors “Better Later Life–He Oranga Kaumātua 2019 to 2034” [6].

Much of the research around AFCC is at a conceptual or pilot stage and focuses on only specific components for AFCC, and the metrics used to assess AFCC interventions are limited [7,8]. Interventions vary and may include the improvement ofin health risk factors for health, positive behavioral lifestyle changes, changes in organizations, and changes in the domains of AFCC, with the majority having a combination of environmental and psychosocial interventions [7]. Examples include safe and friendly housing, transportation, social inclusion and participation, outdoor spaces and buildings, communication, and information [7].

### Smart Cities and Communities, AFCC, and Active Aging

The WHO emphasizes promoting healthy aging in a digital world [9] and identifies loneliness and isolation as critical public health issues requiring urgent intervention [10,11]. The WHO’s extension of the Global Digital Health Strategy to 2027 further underscores the relevance of integrating digital solutions for aging populations worldwide [12].

At its most basic, “smart cities and communities” are cities and communities that are interconnected by digital technology and are data-driven, using AI, to achieve better lives (well-being) for all residents. However, they are not without their own problems [13,14], as the predominant technologies used are the Internet of Things (sensors) and AI [15,16], each of which comes with design, implementation, and sustainability issues. Pedell and Borda [17] combined smart cities, AFCC, and active aging, with 3 of the WHO domains to bring in the digital aspect (Smart) in their conceptual model: a simplified version of the AFCC model by Van Hoof et al [5]. When tested, similar to the authors’ previous research [13,14], they found that digital technology can reduce social isolation, increase social participation, and increase independence. Pedell and Borda [17] suggest that digital technologies may have a wider role to play in AFCC.

### New Zealand Context

Against this international backdrop, this study explores how one New Zealand community is preparing to meet the future well-being needs of its older population. In New Zealand, it is critical that health and well-being are considered under the obligations of Te Tiriti o Waitangi [18] and through a bicultural lens, reflecting Māori perspectives of health. This approach is embodied in the Te Whare Tapa Whā model [19]. Te Whare Tapa Whā defines well-being across 4 interconnected dimensions: physical health (taha tinana), spiritual health (taha wairua), mental/emotional health (taha hinengaro), and family/social health (taha whānau) [19]. These perspectives extend the concept of well-being beyond individual health to include social and community well-being, which are fundamental to Māori, who operate within a collective-based society [20]. This study applies a holistic interpretation of well-being that is consistent with Te Whare Tapa Whā and New Zealand’s bicultural health context.

## Methods

### Study Design

This qualitative exploratory study sought to examine the future well-being needs of older adults residing in a small town and its surrounding rural community, with the aim of informing planning for future service delivery.

### Setting and Context

This study was conducted in a developing rural area in New Zealand’s North Island, historically settled by retired farmers, and characterized by dispersed settlements, limited transport options, and variable internet connectivity. As of 2024, the region had a population of 33,700 people of whom 19.6% were aged ≥65 years [21]. Travel across the region can take approximately 90 minutes. These contextual features create both challenges and opportunities to inform future planning for well-being-related services for an aging population, making older adults a central consideration for this study during the design and analysis stages.

### Participants and Eligibility

The eligibility criteria were community-dwelling older adults born between 1940 and 1960 and residing in the region at the time of data collection. This cohort requirement was set by the project funder.

### Recruitment

Participants were recruited through purposive sampling [22] to ensure diverse perspectives by gender and ethnicity including: male and female participants, Māori, and other non––New Zealand European participants. Recruitment strategies included posters placed in community venues, notices disseminated via local organizations, posts on community Facebook pages, and the researchers’ existing contact networks. These traditional methods of in-person recruiting and using paper-based marketing material were adapted during the recruitment process because the national COVID-19 pandemic lockdown settings prevented in-person contact at the time of data collection. Participation was voluntary. Participants could bring a support person, decline to answer questions, withdraw, and access translation services if required. All participants provided either written or verbal informed consent prior to participation. Verbal consent was an added option for participants due to the restrictions imposed by COVID lockdowns, and returning a scanned informed consent sheet was difficult for some. These recruitment strategies were designed to capture a diverse range of perspectives and ensure cultural inclusivity.

### Data Collection

Data collection occurred between April 5 and May 11, 2022. The project was originally designed to include up to 6 in-person focus groups (a maximum of 40 participants) with older adults born between 1940 and 1960. National COVID-19 pandemic lockdown settings prevented in-person focus groups and necessitated a mixed approach, combining 2 semistructured online focus groups (n=18), three online interviews (n=3), and telephone interviews (n=13). This adaptation of the study’s design also addressed some of the challenges experienced by participants when using digital platforms (such as videoconferencing via Zoom), technical difficulties such as inexperience in using these digital platforms, rural internet connectivity challenges, and preferences for individual interviews. Scanning consent forms was a particular challenge and was addressed using recorded verbal consent.

The adaptive approach taken regarding the initial methodology is consistent with previous rural telehealth research, which shows that flexibility is often required when engaging older populations in digitally constrained contexts [23].

Participants self-rated their confidence in using digital technologies and provided demographic details on age, ethnicity, and gender. Semistructured questions [24] were provided in advance to encourage reflection, a strategy that has proven effective in earlier research [23]. The same questions were used across phone interviews and focus groups for standardization. Four questions, with prompts, structured the sessions (Table 1).

**Table 1.** Questions for focus groups and phone interviews.

Question number	Summary of questions covered
1	How do you see the well-being needs of your community evolving over the next 30 years? What types of community services will be required?
2	Should these services be delivered in- person, digitally, or in a hybrid form?
3	What concerns or barriers might you have in using digital technology to meet the community’s well-being needs? What mitigating factors are required to address these?
4	What benefits could digital technology offer (eg, savings on time, travel, or costs)?

### Data Analysis

An inductive thematic analysis was undertaken [25,26]. All focus groups and phone interviews were audio-recorded, transcribed verbatim, and anonymized, and the data were managed in NVivo (Release 1.6.1; Lumivero). Initial codes were generated, grouped into categories, and refined into overarching themes. Coding decisions and analytic notes were documented to ensure transparency, and themes were reviewed against the full dataset for accuracy. Two researchers coded the data independently and resolved any differences through discussion. The final analysis identified 4 key themes, summarized as the “4 Ss Framework”: services, support, spaces, and social Connection.

### Ethical Considerations

The project followed the Massey University Code of Ethical Conduct for Research, Teaching and Evaluations Involving Human Participants [27] and was judged by peer review as low risk, including suitability from a Māori research perspective. A low-risk notification was recorded with Massey University’s Human Ethics Committee [28]. Māori research ethics considerations were reviewed to ensure cultural appropriateness and responsiveness to Te Tiriti o Waitangi, and the findings were reviewed from a Māori perspective to ensure appropriate interpretation. All participants gave informed consent, and participant involvement was entirely voluntary; privacy was preserved by anonymizing the data, and data were kept secure and confidential as per the university data management guidelines. Participants did not receive financial compensation.

## Results

### Participant Characteristics

A total of 31 older adults aged 61 to 82 years participated, and 45% (n=14) identified as male and 54% (n=17) as female. To allow for birthdays later in the year, the eligibility criteria allowed for 61-year-old participants on the date of data collection.

The majority identified as New Zealand European (n=26, 86%), Māori (n=4, 13%), and “Other” (n=1, 3%), based on the Statistics New Zealand ethnicity classification system [29]. Māori participants were slightly underrepresented when compared to local population data for both the region (18%) and the national population (17%) [30,31]. Findings indicated that 18 (58% (n=18) of participants reported that they were somewhat, or very confident using technology (Figure 1), including the oldest participants, and 63% (n=19) of Manawatū resident participants self-reported as very/ confident or somewhat confident using technology. Table 2 shows the demographic characteristics.

In the findings section, where a participant could be identified with a participant ID, this has been provided. Some quotes are from focus group participants, in which case the relevant focus group participant IDs are provided.

**Table 2.** Demographic characteristics.

Participant ID	Type of contact	Ethnic group	GenderSex	Age (y)	Self-reported level of confidence
P1	Focus group	New Zealand European	Female	71	Very unconfident
P2	Focus group	New Zealand European	Male	73	Somewhat confident
P3	Phone interview	Māori	Female	72	Very confident
P4	Phone interview	New Zealand European	Male	81	Somewhat confident
P5	Focus group	New Zealand European	Male	65	Very confident
P6	Focus group	New Zealand European	Male	70	Very confident
P7	Focus group	Other	Female	76	Very confident
P8	Focus group	New Zealand European	Female	67	Neither
P9	Focus group	New Zealand European	Female	61	Somewhat confident
P10	Focus group	New Zealand European	Male	73	Very confident
P11	Focus group	New Zealand European	Male	80	Neither
P12	Focus group	New Zealand European	Female	82	Somewhat confident
P13	Focus group	NZENew Zealand European	Male	70	Somewhat confident
P14	Focus group	New Zealand European	Female	66	Somewhat confident
P15	Focus group	New Zealand	Female	67	Neither
P16	Focus group	New Zealand	Male	70	Neither
P17	Focus group	New Zealand	Male	69	Neither
P18	Focus group	New Zealand	Female	66	Very unconfident
P19	Focus group	New Zealand	Female	77	Very unconfident
P20	Focus group	New Zealand	Female	82	Somewhat confident
P21	Phone interview	New Zealand	Male	73	Somewhat confident
P22	Phone interview	New Zealand	Female	70	Somewhat confident
P23	Phone interview	New Zealand	Female	70	Neither
P24	Phone interview	Māori	Female	61	Very confident
P25	Phone interview	New Zealand	Male	64	Neither
P26	Phone interview	New Zealand	Female	74	Neither
P27	Phone interview	New Zealand	Male	61	Very confident
P28	Phone interview	New Zealand	Male	74	Somewhat unconfident
P29	Phone interview	New Zealand	Female	65	Somewhat confident
P30	Phone interview	Māori	Male	70	Neither
P31	Phone interview	Māori	Female	65	Somewhat confident

### Thematic Findings: The 4 Ss Framework

Thematic analysis identified 4 interrelated domains of well-being—services, support, spaces, and social connection—which together form the 4 Ss Framework. Table 3 presents these overarching themes and their associated subthemes. These themes reflect priorities identified by participants when considering what older adults will need to age well over the next 30 years, as required by the project funder.

**Table 3.** Themes and subthemes (4 Ss Framework) identified by participants.

Theme	Subtheme
Services	Transport, housing, health care, and communication
Support	Digital technology, connectivity, safety and security, information
Spaces	Existing facilities, purpose-built social spaces, technology hubs, and hybrid (digital and physical spaces)
Social connection	In-person interactions and socializing, loneliness and isolation, feeling part of the community, Te Ao Māori, and connection

### Theme 1: Services

Participants consistently emphasized the importance of services that sustain independence and well-being, particularly transport, health care, housing, and communication.

#### Transport

Transport emerged as the most pressing concern. Rural participants described high fuel costs, limited public transport, and mobility barriers to reaching bus stops. Some worried about dependence on family: *“*I can get lifts, but I don’t want to be dependent on others*”* (P1). Alternatives suggested included subsidized taxis, shuttles, and mobility scooters. Participants considered autonomous (self-driving) cars as viable future transport options, where one said, *“*I’m looking forward to autonomous cars when I stop driving*”* (P6). Taxi services were considered better for social interaction and more personal than taking the bus.

The prospect of losing a driver’s license generated considerable anxiety: “I am dreading what happens if I can’t drive*”* (P2). Participants linked this to reduced social interaction and an increased risk of isolation and loneliness: *“*I hate the idea that we can’t go out at night” (P3).

#### Housing

Housing affordability and limited options constrained the ability to relocate closer to services or family,: “Housing developments are currently targeted to those who are well off financially” (P21). Mixed developments that combine public, private, intergenerational, and sheltered housing were widely supported. *“*The actual village has young people and children and families and workers and retirees and unemployed, that is the sort of caring community I would like to see for the ageing population” (P3).

Smart home technology in housing was generally regarded positively. Different levels of familiarity with smart home technology were recognized by participants,; *“*some people in NZ already have semi-intelligent houses, already know how to use Alexa and technological gadgets” (P5), while another acknowledged that *“*people that haven’t grown up with [it] are going to need the reassurance that they can use these things in the next 30 years” (P6).

#### Health Care

Health care was another key issue. Concerns were raised about limited local providers, long wait times, and travel distances for rural residents. These concerns ran the full gamut of health care, including emergency care, acute care, long-term care, disability support, including support for low vision and deaf communities, mobility, and dementia care. Changes discussed included walk-in clinics, different opening times, and reduced waiting times, with one participant likening the current health service to “a very slow train crash” (P3). Some participants recollected the district nurses’ home visits and expressed concern about their decline. Cost was raised as a general issue, (*“*Living off the pension now is prohibitive, power, phone, insurance, ….” ([P27]) and specifically around private health insurance (*“*By the time you need your health insurance, you can’t afford it, it is too expensive” ([P3]).

Telehealth (Tthe interpretation of Telehealth applied here is “the use of digital technology to deliver health care when participants are separated by distance and/or time.” [32]) was valued because it gives “the ability for medical practitioners to see into your life where you are more relaxed” (P7). However, downsides raised about telehealth included: confidence, digital skills, and access to technology or connectivity. Participants expressed a clear preference to have the option of in-person consultations and acknowledged that telehealth could not replace all aspects of health care. “You can see a dead cow out there on a drone, or a cow lying down, you don’t know what’s wrong with it until you go up and see it*”* (P30).

Delivery of medications by drone (P9, and P24) was of particular interest to rural participants because accessing the local pharmacist required lengthy road trips. Unlike other rural areas, where the local rural bus can deliver medications (and shopping), many rural residents in the study area have no option but to drive to the local pharmacy in town.

One participant with low vision commented: *“*For our mental health, one of the main things is we need someone who we can connect with, who understands us as an individual, and we feel is on our side, that we can say, ‘this is what I am doing, how do I go about it’*”* (P29). Health and loneliness can be intertwined, and for example, “Isolated people feel pain more than those socially connected” (P3).

#### Communication Gaps

Communication gaps were seen as a significant issue, especially as both rural and urban populations increase,: *“*the bigger the place gets, the worse the communication gets” (P5). Many participants lacked timely information about services, events, or smaller community groups, constraining their ability to access services, which would improve their social connection. This was exacerbated for people who were isolated, new to the community, or did not have English as their first language. While community groups exist, *“*there is no overarching facilitation of these groups spreading the word*”* (P7), illustrating the opportunity for community navigators to share information to improve social connection.

Digital tools provided opportunities to communicate, but barriers such as cost, skills, and access excluded some older adults: *“*A large proportion of the population can’t communicate using tech, or don’t want to*”* (P2), and one participant commented that *“*There was an online survey for the rural bus service, I got it when it was sent out, but couldn’t save it on the computer–not like pinning it on the fridge with a magnet*”* (P1). On the other hand, digital technology assisted with communication,: *“*Technology allows people to be in contact with friends who live far away” and “translation devices help people connect despite the language barrier” (P22).

### Theme 2: Support

Discussions about support centered on the importance of digital literacy, reliable connectivity, support for safety both online and personal safety, and the developing skills around information management.

#### Digital Technology

Participants recognized the increasing integration of technology in daily life (including as a tool for sharing information) and acknowledged its advantages, including online banking, virtual travel, and telehealth. However, participants also voiced strong concerns about being excluded due to limited access, affordability, or digital literacy, noting that *“*as time goes on people will have to accept technology*”* (P11-P20). The rapid rate at which digital technology is changing was considered a barrier*,: “*This is why some people give up, and then they become isolated*”* (P5).

Some participants reported using devices to connect with family overseas or for entertainment. Barriers to the uptake of digital technology by older adults included low confidence and digital skills,: *“*Older people are feeling cut out because they aren’t technologically literate” (P11-P20), fear of scams, and associated costs with *“*trust is a huge barrier to people using technology*”* (P21).

Support groups are one way of addressing low confidence and digital skills: *“*with the Blind Low Vision [support group], we have been learning how to use Alexa and Siri, but once you get to people age 65‐70, anything digital is hard work if you don’t understand the workings” (P28) and “I also have hearing problems, which a lot of people probably have both problems with vision and deafness” (P29). P29 added that, *“*the Blind Low Vision person came over and showed me how to use Siri on my phone, I now ask the phone for location directions.”

#### Connectivity bBarriers

Connectivity barriers included unreliable rural internet and phone coverage, unclear pricing, and restrictive data caps, which exacerbated digital exclusion for older adults. Several participants stressed that knowing about and using relevant apps was as important as the infrastructure itself,: *“*the online communication will only work if people know about the apps, have the apps, can use these and have access to the technology” (P9). Unreliable coverage was perceived as a barrier to engagement: *“*You can’t give your mobile number out, because no one can contact you when you get home*”* (P8) (due to variable coverage).

#### Safety and Security Concern

Safety and security concerns were a broad topic, including housing security, especially when living alone, feeling safe when out and about or at home (not just from physical attacks but from falling or injuring themselves), trusting people who came to the house such as trades people, and so on. Participants suggested that pedestrian crossings require level access and “Cross Now” audio signals that are loud enough for people who are deaf or have hearing loss to improve their safety.

Some participants did not know how to improve their internet connectivity and often worried about whether they had completed their online banking or email transactions successfully. This led to frustration and an increased fear around using the internet for certain activities. Concerns heightened when phishing was discussed, because participants worried about online fraud and their ability to recognize it,: *“*I cancelled my card and started from scratch again, my children helped me with that. What was scary was that the person on the phone [from the bank] said, ‘you would be surprised how often that happens*.’* I don’t know what we can do about tightening up security on devices” (P23). In addition, there can be a level of discomfort seeking help with sensitive digital tasks such as banking,: *“*Older people may not want strangers to help with certain aspects of technology” (P22).

#### Information

Information, and in particular, digital literacy, emerged as a recurring theme. As more services move online, participants are concerned about their ability to search, verify, and store information effectively,: “if older adults are active in the community, they will get information but if [they are] at home they will be isolated and not get the information*”* (P27). Concern about managing the “social media afterlife” (P5) following death highlighted the need for better guidance. Practical, community-based training to improve digital skills was strongly supported.

### Theme 3: Spaces

Spaces for social connection and well-being were considered essential, with a preference for adapting existing facilities over constructing new ones to enable in-person interactions.

#### Existing Facilities

Community venues, such as halls, cafés, and churches, were described as underused yet valuable resources: *“*We don’t need new buildings, but we should utilisze what we have” (P10, P27).

#### Purpose-Built Social Spaces

On the other hand, building new social spaces designed to accommodate the needs of the community by being age-friendly and accessible was considered necessary–*“*We need more community buildings in place” (P4, P27). Barriers identified included cost, compliance requirements, and physical accessibility challenges. Participants expressed a preference for casual, spontaneous use of spaces, particularly cafés as community hubs.

#### Technology hHubs

Technology hubs co-located in libraries or health care centers were recognized as preferred spaces for supporting informal learning and scheduled training. One participant identified that *“*The library could encourage people to come together–they tend to do this with other groups, but not necessarily older people” (P4). These shared spaces could also support tasks such as logging into patient portals by providing technological support.

#### Hybrid Spaces

Hybrid spaces that allow both in-person and online participation were considered particularly valuable for individuals with low vision, mobility, or transport challenges. It was proposed that delivering introductory in-person training (eg, teaching videoconferencing skills) would increase older adults’ confidence and willingness to engage online. As one participant said, *“*We could have an in-person session initially to show us all how to use Zoom, so we would feel more comfortable” (P2).

### Theme 4: Social Connection

Social connection (also known as social connectedness) was consistently identified as a cornerstone of well-being.

#### In-Person Interactions and Socializing

In-person interactions and socializing had the most impact on well-being in all the focus groups and interviews. Most conversations concluded with a discussion highlighting the importance of socializing in person. In-person connection was perceived as irreplaceable, with digital tools valued only as supplementary supports. As one participant explained, “We are lucky in that we all belong to each other, we are whakapapa, and we can talk to each other, but in other communities that is not the case where they are diverse” (P24).

Everyday connections through clubs, volunteering, and informal gatherings provided purpose and belonging, with some attending clubs *“*for the interaction” (P10) rather than for the activity itself. Intergenerational contact, whether with family or through community programs, was described as enriching and valuable for participants,: “Mixing with the local youth is a win” (P10).

While digital platforms supported social ties during the COVID-19 lockdowns, participants agreed that physical spaces and in-person connections were essential to their well-being. Participants were clear that virtual options should complement, not replace, in-person contact.

#### Loneliness and iIsolation

Loneliness and isolation were attributed to a lack of social connection in the community, limited inclusive spaces, or inadequate access to digital technology, and were further linked to limited mobility or a recent relocation to the region.

As one participant stated, *“*without a Tablet or Smart phone, older adults miss out on the connection, and struggle to get information” (P27). New residents, in particular, described difficulties integrating and, at times, feeling unwelcome in established communities. Participants were very keen to explore social prescribing and community well-being navigators, both strategies deployed in New Zealand and overseas to increase social contact and connection, *“*if there was a connector that would help” (P7).

Another participant commented, *“*For a lot of us, it is family, I have quite a few friends who are on their own, and it is hard. I see that digital can help, but there is nothing like a person who doesn’t look like they are in a hurry to get to the next job – they have time” (P29).

#### Not Part of a Community

Participants were concerned that newcomers from urban areas often preferred to keep to themselves and often worked from home or commuted to larger urban centers, coming home to relax. They felt that these new residents did not always recognize the importance of social and community connection and were therefore less likely to engage in community activities.

Even existing residents can feel disconnected from the wider community, with their only contact being through social services: *“*I deliver meals on wheels, and it’s obvious that a lot of people have no contact other than the person bringing their meal” (P23).

#### Te Ao Māori and Connection

Māori participants emphasized the value of cultural connection*—“*I would be more comfortable talking to you face to face, as opposed to this (Zoom)” (P30)—including the central role of marae (Māori meeting places), which operates as multifunctional hubs that provide cultural, social, and increasingly digital engagement. A number of marae now offer fast internet, enabling them to serve as hybrid spaces, as one participant noted: “*Y*es, there is Wi-Fi on the marae…but not in our houses” (P24). This can also provide support in using technology, with one participant observing, “Technology for our community, we have old people out here (The Valley), they don’t even know how to operate a mobile phone” (P31).

## Discussion

### Principal Findings

The 4 Ss Framework (Table 3) provides a practical lens for understanding future well-being priorities in a regional older adult population by highlighting 4 priority areas for future planning for age-friendly, inclusive communities: services, support, spaces, and social connection. Notably, participants described well-being as relational and community-based, reflecting Māori perspectives that emphasize collective rather than solely individual determinants of health. Social and community well-being emerged not only as one of the 4 Ss but also as a determinant that influences access to services, digital engagement, and the use of physical spaces.

The approach proposed aligns with internationally recognized social determinants of health and well-being [2] and the WHO dimensions of active aging [3]. The thematic findings presented here also reflect the models of Van Hoof et al [5] and Pedell and Borda [17], which emphasize the interplay between environments (spaces), service access, and social participation in shaping aging experiences. Despite reporting confidence with using digital technologies, participants’ difficulties in participating online reflected limited skills and low trust in digital systems, illustrating a gap between perceived and actual digital literacy.

These findings confirm and extend prior research demonstrating links between social isolation and poorer health outcomes [33] that telehealth faces persistent equity barriers [34] and the need to co-design housing and transport to support aging in place [13]. Alignment with global and national policies on active and healthy aging, the WHO’s Global Network for AFCC [35] and New Zealand’s Healthy Aging Strategy [36], is also evident due to their emphasis on transport, housing, communication, and participation as the foundation of age-friendly environments. Similarly, New Zealand’s Better Later Life–He Oranga Kaumātua 2019 to 2034 strategy [6] sets a vision to enable participation and value contributions as people age. The alignment of this study’s findings within a wider policy context highlights their relevance for future planning.

Most participants commented that younger generations are more digitally literate than older generations, suggesting that communities are experiencing a transition phase, with any perceived lack of digital skills primarily affecting today’s older adults and possibly the subsequent generation.

### Services

Participants highlighted concerns related to financial insecurity, isolation and loneliness, safety and security, stress and anxiety, and limited access to social services. Challenges in accessing social services can disproportionately affect people who are deaf or have hearing loss, as well as those with low vision, especially those without standard forms of identification, such as a driving license. Looking to the future, essential foundations for aging well in place included the importance of reliable access to health care, housing, transport, and information.

Enabling access to reliable and safe transport supports social participation and inclusion because the older adults’ ability to engage in- person is contingent on accessible mobility options, an essential principle in universal design and age-friendly environments [37,38]. Autonomous (self-driving) cars were considered viable and realistic future options, either as owner-user or taxi-based models, and the deployment of these technologies is already emerging internationally [39-41]. Shu and Woo [42] anticipate that autonomous mobility may help reduce older adults’ social and geographic isolation by overcoming transportation barriers. These access challenges ultimately constrained opportunities for social participation and inclusion, demonstrating the importance of treating social well-being as a core service outcome.

Health care concerns focused on access, long waits, and rural service gaps. Participants viewed telehealth and wearable technologies as useful in supporting in-person care within a hybrid delivery model. However, they emphasized that telehealth could not replace all aspects of health care and that retaining the option for face-to-face consultations remains essential for many older adults [23]. This finding reflects global evidence that the effectiveness of telehealth depends on confidence, digital skills, technology availability, and reliable connectivity [34,43-45]. Social isolation, and its impact on health, emerged as a key concern for participants, reinforcing international findings that social isolation can exacerbate pain and reduce participation in daily activities [33].

While participants in this study did not raise cost as a specific barrier to health care, this may be due to the population sampled, as several reported holding private health insurance, although many found it too costly to maintain. Despite universal health coverage being the predominant funding model in New Zealand, around 33% of the population buy additional private health insurance at some point [46]. However, in New Zealand’s *Better Later Life–He Oranga Kaumātua 2019 to 2034* strategy, cost was clearly identified by older adults as a barrier to health care and well-being, for example, in relation to visiting the doctor and dentist [6].

Housing affordability and diversity of types of housing were equally pressing needs, with participants supporting smart home technologies and universal design features to enable aging in place. Smart home sensors can support safety and daily activities, enabling more older adults to age in place, which is associated with better health outcomes than relocating to assisted living [13,14]. International evidence identifies that early integration of universal design reduces costs and supports disability-inclusive housing [13,14]. Jacques [47] has argued that any new house has a 60% probability of accommodating someone with a disability during the lifespan of that house. Transport and housing are interlinked, with the location and design of new developments requiring integration with accessible public and active transport options to avoid environments that limit older adults’ mobility, social participation, and inclusion and thereby creatinge islands of isolation [48].

The transition from print to digital was seen as a significant communication challenge that risks excluding those lacking digital access or skills, thus increasing social isolation [49]. This reflects broader international concerns that digital transitions can exacerbate inequalities in information access, leading to digital exclusion for some communities [50,51]. Seifert et al [52] found that older adults often feel excluded when essential communication moves online without alternative nondigital alternatives. Vaca-Benavides et al [53] also caution that digital transitions in health and social care services can exclude older adults who struggle with digital confidence, access, connectivity, or support.

### Support

Trust in digital technology, in the systems that support it, and in participants’ own ability to use it emerged as a significant concern and presented issues with information access. Barriers identified included cost, the rapid pace of technological change, and limited training opportunities. One option to address this is to provide community well-being navigators (through local libraries) to support older adults with digital literacy and increase their confidence in the use of digital technologies [54]. Vercruyssen et al [55] observed that for older adults with minimal or no prior experience with digital technology, acquiring even the most basic or entry-level digital literacy skills is difficult.

Internet and phone connectivity issues, experienced particularly by participants living in rural areas, compounded these barriers to equitable digital access. Even in areas with theoretical internet coverage, participants experienced unreliable connections, consistent with national and global studies highlighting the last mile of the digital divide [23,56].

The participants also raised safety and security concerns about the use of digital technologies, including online risks (eg, fraud and identity theft) and physical vulnerabilities. Technologies such as alarms and GPS trackers were predominantly welcomed, especially for dementia care; however, participants’ low levels of trust presented barriers to digital engagement. Kebede et al [57] reported that safety and digital confidence issues represent key barriers to digital engagement, particularly in relation to web-based digital technologies. The ability to access digital information and community services was considered essential to well-being [43], because unreliable access increased the risk of social isolation.

Digital literacy is essential for civic participation and safety. Older adults are at risk of digital exclusion without targeted support [55]. Digital skills therefore support more than information access; they enable older adults’ civic participation and social connection, which is critical to community well-being. Seifert et al [52] described a “double burden of exclusion” affecting older adults who face both digital and social exclusion. Other research illustrates that affordability, trust, and skills gaps continue to drive digital exclusion among older adults [58,59], mirroring the findings of this study.

### Spaces

Accessible, inclusive, and multifunctional community spaces that support social and recreational activities were considered essential for well-being. One key finding was participants’ clear preference for reusing existing facilities as social spaces, such as community halls, cafés, and churches, rather than building new ones, which is consistent with international research. Liddle et al [60] recognized the importance of making better use of existing spaces to improve social connections and considered how to repurpose them. Barriers identified to repurposing shared spaces included cost, compliance requirements, and accessibility challenges. Integrating age-friendly universal design into both housing and public infrastructure could reduce these barriers and promote independence, because urban design influences opportunities for social engagement and inclusion [61]. Physical places that enable social connection were regarded as essential infrastructure for community well-being, not merely a venue for activities. With older adults’ strong preference for in-person interaction, it is essential that we retain and repurpose existing spaces to support their well-being. While digital technology can complement these physical spaces, it cannot substitute for them, particularly for those at risk of digital exclusion.

### Social Connection

Social and community well-being emerged as a key determinant of healthy aging, influencing participation, belonging, and independence across all aspects of daily life. Social interaction, particularly in-person connection, was consistently described as essential to well-being, especially for those with low vision and hearing loss. A lack of social connection was perceived to contribute to loneliness and isolation, negatively impacting mental health. International research has similarly identified that increasing opportunities for in-person social contact can reduce loneliness and improve well-being in older adults, consistent with the findings of this study [61].

Participants emphasized the value of social prescribing [62] and community well-being navigators [54], both of which are emerging as strategies in New Zealand and internationally to increase social contact and reduce isolation. Māori participants emphasized the importance of marae as multifunctional hubs, reinforcing indigenous models of well-being where holistic dimensions are interconnected. The exploration of marae-based approaches, and similar collective spaces, is recommended to strengthen belonging and intergenerational ties to support social connection. Participants expressed a clear preference for approaches that combine digital access with ongoing in-person contact, such as hybrid models, reflecting findings internationally that found face-to-face interaction crucial for older adults’ well-being [63-65].

In summary, these findings suggest that future age-friendly planning must prioritize social and community well-being outcomes, ensuring that investments in services, digital inclusion, and public spaces strengthen older adults’ ability to connect with others and participate fully in community life.

### Recommendations

The 4 Ss Framework is proposed as a planning framework for future age-friendly, inclusive communities: services, support, spaces, and social connection. It can be used to assess current opportunities, identify gaps in existing opportunities, and thus inform future planning. Using the framework, the authors propose a set of actionable recommendations to guide planning for age-friendly regional communities over the next 30 years. Table 4 summarizes these recommendations, offering actionable guidance for service providers, policymakers, and community organizations. This is also an example of how a community could use the framework for its future age-friendly planning.

These recommendations, drawing on the 4 Ss Framework, are designed to inform future planning for age-friendly regional communities over the next 30 years, as well as corresponding policy and practice implications. The findings highlight the importance of hybrid service models, digital inclusion, universal design, and social connection and emphasize the importance of embedding aging perspectives in policy and planning. Finally, most participants commented that younger generations are more digitally literate than they are, suggesting that communities are experiencing a transition phase , with any perceived lack of digital skills primarily affecting today’s older adults, and possibly, the subsequent generation.

The future offers many opportunities for supporting the well-being of individuals and communities, and the findings of this project are supported by existing models of wellness. Using the models and strategies in the literature, highlighted by this thematic analysis and the Te Whare Tapa Whā model, it is possible to create a matrix to direct planning and evaluation of interventions and initiatives to improve wellness in age-friendly communities that meet Te Tiriti o Waitangi obligations and align with New Zealand healthy aging strategies. Table 5 shows an example of such a matrix, with potential initiatives available in New Zealand.

**Table 4.** Recommendations for supporting older adults’ well-being in regional communities.

Theme (4 Ss Framework)	Recommendation	Rationale/evidence
Services	Prioritize hybrid service models that combine digital and in-person delivery	Digital tools are useful but cannot replace in-person interaction; hybrid models improve access and support well-being
Support	Reenvisage community spaces as multifunctional hubs for digital training, service access, and social connection	Lack of digital skills and trust creates exclusion, training in familiar settings, builds confidence and inclusion
Spaces	Apply universal design to housing, public infrastructure, and transport	Universal design ensures accessibility; improved transport access enables participation, independence, and aging well in place
Social connection	Establish well-being navigator roles to support older adults in accessing services	Limited trust in technology is a barrier; navigators build trust, reduce isolation, and strengthen community networks

**Table 5.** Proposed matrix for wellness interventions, with some examples.

Examples of wellness interventions	Services	Skills	Spaces	Social connectedness
Physical health/te taha tinana	Meals on wheels	Fitbits, waka ama	Gyms, safe pavements	Social prescribing
Spiritual health/te taha wairua (sense of meaning and purpose)	Older adults working with youth	Virtual reality journeys	Marae, museum	Kai Hub, Kapa haka
Mental or emotional health/te taha hinengaro	Well-being navigators	Mobile banking	Safe public gardens	Repair cafes and Menzshed
Family/te taha whānau (social well-being)	Free hearing aids	SeniorNet	Library	Skinny Jump, social media

### Limitations

This study was based on a small regional population in New Zealand and relied on self-reported experiences, both of which are common limitations in focus group studies. The COVID-19 public health restrictions and national lockdowns at the time shaped recruitment and influenced preferences for individual interviews over group discussions, as well as limitations to completing documentation such as consent forms. As the number of individual interviews grew, fewer new ideas emerged; however, determining data saturation remains contentious, with varying definitions across qualitative research. Therefore, caution is required when applying the findings beyond the study location.

### Future Research

Opportunities for further studies include testing the 4 Ss Framework in other settings, for example, with older adults with low vision examining postpandemic digital adoption and evaluating the effectiveness of social prescribing and well-being navigator roles in facilitating digital engagement. The authors are not qualified to speak on Te Ao Māori but suggest a closer exploration of how marae support, and can be supported, to enhance the well-being of Māori, and indeed other Indigenous peoples, recognizing not only the importance of maintaining tradition and belonging, but also as to how it can be applied to non-Māori and other non-Indigenous populations.

### Conclusions

The future presents significant opportunities to enhance the well-being of older adults and communities through targeted, inclusive, and innovative approaches. Building on the thematic analysis and existing strategies, this study proposes a practical framework (the 4 Ss Framework) to guide future planning for age-friendly communities. As a methodology, the 4 Ss Framework provides a structured approach to anticipating the future well-being needs of older adults to support them to age well and age in place. By translating the lived experiences of older adults into an actionable framework, this study contributes to local and international efforts to create inclusive, resilient, and age-friendly communities. The 4 Ss Framework reflects the priorities of the WHO Global Network for AFCC, the UN Decade of Healthy Ageing, and OECD (Organisation for Economic Co-operation and Development) well-being agendas, offering a transferable approach designed as a tool to support and strengthen the future well-being of communities.

## Abbreviations

AFCC: Age-Friendly Cities and Communities
OECD: Organisation for Economic Co-operation and Development
WHO: World Health Organization
